# Structural comparison of protiated, H/D-exchanged and deuterated human carbonic anhydrase IX

**DOI:** 10.1107/S2059798319010027

**Published:** 2019-08-22

**Authors:** K. Koruza, B. Lafumat, M. Nyblom, B. P. Mahon, W. Knecht, R. McKenna, S. Z. Fisher

**Affiliations:** a Department of Biology and Lund Protein Production Platform, Lund University, Sölvegatan 35, 223 62 Lund, Sweden; b Department of Biochemistry and Molecular Biology, University of Florida, Gainesville, FL 32610, USA; c Scientific Activities Division, European Spallation Source ERIC, Odarslövsvägen 113, 224 84 Lund, Sweden

**Keywords:** carbonic anhydrase, proton transfer, X-ray crystallography, carbonic anhydrase IX, neutron protein crystallography, perdeuteration, proton transfer

## Abstract

Human carbonic anhydrase IX (CA IX) expression is upregulated in hypoxic solid tumours, promoting cell survival and metastasis. This observation has made CA IX a target for the development of CA isoform-selective inhibitors. To enable structural studies of CA IX–inhibitor complexes using X-ray and neutron crystallography, a CA IX surface variant (CA IX_SV_; the catalytic domain with six surface amino-acid substitutions) has been developed that can be routinely crystallized. Here, the preparation of protiated (H/H), H/D-exchanged (H/D) and deuterated (D/D) CA IX_SV_ for crystallographic studies and their structural comparison are described. Four CA IX_SV_ X-ray crystal structures are compared: two H/H crystal forms, an H/D crystal form and a D/D crystal form. The overall active-site organization in each version is essentially the same, with only minor positional changes in active-site solvent, which may be owing to deuteration and/or resolution differences. Analysis of the crystal contacts and packing reveals different arrangements of CA IX_SV_ compared with previous reports. To our knowledge, this is the first report comparing three different deuterium-labelled crystal structures of the same protein, marking an important step in validating the active-site structure of CA IX_SV_ for neutron protein crystallography.

## Abstract 2

In this work, the X-ray crystal structures of four different deuterium-labelled versions of a surface variant of human carbonic anhydrase IX are compared and discussed. The results show that the overall structure and active-site organization of each version are essentially the same, paving the way for future neutron protein crystallography studies.

## 1. Background   

Carbonic anhydrases (CAs) are zinc-containing metallo­enzymes that catalyze the reversible hydration of CO_2_ to form HCO_3_^−^ and H^+^. The first step of the reaction in the hydration direction results in a water molecule bound to the zinc that has to be deprotonated to generate a zinc-bound OH^−^ for subsequent reactions. The excess H^+^ is transported via an ordered hydrogen-bonded water network to a proton-shuttling residue, His64 (CA II numbering), that ultimately delivers the H^+^ to the bulk solvent (Coleman, 1967 ▸; Silverman & McKenna, 2007 ▸). Numerous X-ray and neutron crystallo­graphic studies have shed light on the catalytic mechanism of CA and have provided insights into the details of the finely tuned active site that supports CO_2_ hydration and rate-limiting proton transfer at very high rates (*k*_cat_ = 10^6^ s^−1^; Kim *et al.*, 2016 ▸; Fisher *et al.*, 2007 ▸, 2011 ▸; Domsic & McKenna, 2010 ▸).

There are 15 expressed CA isoforms in humans that show diversity in expression between tissues and organs, supporting a range of physiological functions. One of the isoforms, CA IX, has limited expression in healthy tissues but is upregulated in aggressive tumours, with its expression being controlled by hypoxia (Pastorek & Pastorekova, 2015 ▸). CA IX is a multidomain membrane-bound protein, with its catalytic CA domain facing extracellularly (Langella *et al.*, 2018 ▸; Alterio *et al.*, 2009 ▸). CA IX upregulation is part of a number of cancer-cell adaptions to hypoxia and is thought to occur in response to the lowering of pH in the cancer extracellular environment. The tumour pH environment is adapted from a physiological pH of 7.4 to as low as 6.0 (Mahon *et al.*, 2015 ▸; Pastorek & Pastorekova, 2015 ▸). This acidification promotes metastasis, most likely through protease activation and degradation of the extracellular matrix. A meta-study of patient outcomes showed very poor patient prognosis when positive for CA IX expression (Kuijk *et al.*, 2016 ▸). For these reasons CA IX is a promising target for cancer detection and therapy, but high sequence conservation among human CAs (30–80% amino-acid identity) results in indiscriminate binding of the currently available regime of clinically used CA inhibitors. Hence, there is a recognized need to develop isoform-specific inhibitors that inhibit CA IX strongly while ideally not inhibiting the other CAs (Pinard *et al.*, 2015 ▸; Mahon *et al.*, 2015 ▸).

Recent work by Mahon and coworkers reported the biophysical characterization and first X-ray crystal structure of a surface-modified variant of CA IX (CA IX_SV_; Mahon *et al.*, 2016 ▸). CA IX_SV_ contains only the catalytic domain of CA IX, with the intracellular, transmembrane and PG domains removed. In addition to truncating the full-length protein, six surface mutations (C174S, L180S, M360S, A210K, A258K and F259Y) were also introduced. These were chosen to remove a disulfide bond, to reduce surface hydrophobicity and to promote crystallization based on the crystal contacts in CA II (Mahon *et al.*, 2016 ▸). The native, full-length protein is produced in insect cells in low yields, and is not stable or very soluble (Alterio *et al.*, 2009 ▸). In this manuscript, and in the models deposited in the PDB, we use CA IX numbering. All residues mentioned in the text, tables and figures are for CA IX.

Endogenous CA IX is functional in a lower pH environment compared with other CAs. As such, Mahon and co­workers measured the thermal stability and catalytic parameters of CA IX under different pH conditions and demonstrated its adaptation to low pH, giving rise to its structural and functional stability at pH values as low as 5.0. The p*K*_a_ of the H^+^ donor and acceptor groups in the active site are also decreased compared with CA II, indicating an ability to retain enzymatic activity at a pH of ∼6 (Mahon *et al.*, 2016 ▸).

Owing to the need for the development of CA isoform-specific inhibitors against CA IX, and to gain a deeper understanding of its active-site architecture, our goal is to utilize joint neutron and X-ray structures of CA IX_SV_ alone and in inhibitor complexes to fine-tune compounds to preferentially bind CA IX over CA II (Langan & Chen, 2013 ▸; Aggarwal *et al.*, 2013 ▸; Kovalevsky *et al.*, 2018 ▸). Previous neutron crystallographic studies of CA II in complex with clinically used inhibitors (for example brinzolamide, ethoxzolamide and acetazolamide) revealed the role and importance of water and hydrogen bonds in mediating ligand-binding interactions (Kovalevsky *et al.*, 2018 ▸; Fisher *et al.*, 2012 ▸). In preparation for future neutron protein crystallo­graphic studies of experimental inhibitors binding to CA IX, we expressed unlabelled CA IX (H/H CA IX_SV_) and performed H/D exchange (H/D CA IX_SV_) on preformed crystals. We also expressed deuterated protein (D/D CA IX_SV_) for crystallization (Fisher *et al.*, 2014 ▸; Koruza, Lafumat, Végvári *et al.*, 2018 ▸; Blakeley *et al.*, 2015 ▸).

There are several studies that have compared the properties, activities and X-ray crystal structures of unlabelled (H/H) and perdeuterated (D/D) versions of the same protein: cholesterol oxidase, haloalkane dehalogenase and arginase I (Golden *et al.*, 2015 ▸; Liu *et al.*, 2007 ▸; Di Costanzo *et al.*, 2007 ▸). These studies all showed minimal structural effects owing to perdeuteration (D/D). However, we could not find studies in which X-ray crystal structures of H/D-exchanged versions were also included in the analysis. As H/D exchange is the most commonly used form of deuterium labelling for neutron protein crystallographic studies, it is important to determine and verify whether labelling by itself has any effect on the crystal structure. Here, we present a comparative structural analysis of three different isotopically labelled (H/H, H/D and D/D) forms of CA IX_SV_ and show that the overall fold and the active-site side-chain conformations are mostly unaffected. However, there are some subtle changes in solvent positioning that may be owing to deuteration effects and/or to differences in the resolutions of the structure determinations. We also analyzed the crystallographic monomer-to-monomer contacts and packing for different space groups of CA IX_SV_ compared with previous reports.

## 2. Materials and methods   

In this study, we use three designations to indicate protiated, H/D-exchanged and deuterated status: H/H means protiated protein in protiated buffer, H/D refers to protiated protein that was subjected to vapour H/D exchange after crystallization and D/D is deuterated protein that was purified in protiated buffers and then subjected to back-exchange in solution to recover any lost D atoms. For all studies we used a construct created by Mahon *et al.* (2016 ▸) containing the catalytic domain of CA IX with six surface mutations introduced (C174S, L180S, M360S, A210K, A258K and F259Y) that was engineered to facilitate crystallization (CA IX_SV_).

### 2.1. Expression and purification of protiated CA IX_SV_   

CA IX_SV_ production has been described in detail elsewhere (Koruza, Lafumat, Végvári *et al.*, 2018 ▸; Mahon *et al.*, 2016 ▸). Briefly, CA IX_SV_ was expressed in *Escherichia coli* BL21 (DE3) cells under kanamycin selection (final concentration of 50 µg ml^−1^) in a shaking incubator at 37°C. The cells were grown to an OD_600_ of ∼1.0 and expression was induced by the addition of 1 m*M* isopropyl β-d-1-thiogalactopyranoside (IPTG) in the presence of 1 m*M* ZnSO_4_. After 4 h the cells were harvested by centrifugation (5000*g* for 20 min) and the cell pellets were frozen at −20°C. The cell pellets were lysed by thawing at room temperature in 0.2 *M* sodium sulfate, Tris–HCl pH 9 and then stirring in the cold room for ∼3 h in the presence of 20 mg lysozyme and 1 mg DNaseI. Clarified lysates were prepared by centrifugation at 50 000*g* for 60 min at 4°C. Affinity chromatography using *p*-aminomethylbenzenesulfonamide resin (Sigma–Aldrich) (wash buffer 1, 0.2 *M* sodium sulfate, Tris–HCl pH 9; wash buffer 2, 0.2 *M* sodium sulfate, Tris–HCl pH 7; elution buffer, 0.4 *M* sodium azide, 50 m*M* Tris–HCl pH 7.8) was followed by size-exclusion chromatography (50 m*M* Tris–HCl pH 7.8, 100 m*M* NaCl). CA IX_SV_ elutes from the size-exclusion column in two peaks corresponding to dimeric and monomeric forms (Koruza, Lafumat, Végvári *et al.*, 2018 ▸).

Peak fractions corresponding to monomeric CA IX_sv_ were pooled and concentrated using Amicon Ultra Centrifugal Filter Units (Merck) with a molecular-weight cutoff of 10 kDa and were analyzed by sodium dodecyl sulfate–polyacrylamide gel electrophoresis (SDS–PAGE) to estimate their purity. The protein was concentrated to a final concentration of 17 mg ml^−1^ for crystallization.

### 2.2. Expression and purification of deuterated CA IX_SV_   

Deuterated CA IX_SV_ was expressed in *E. coli* BL21 (DE3) cells according to a protocol described elsewhere (Koruza, Lafumat, Végvári *et al.*, 2018 ▸). Briefly, cells were pre-grown in LB Broth (Miller) (Difco) at 37°C. The growth medium was then exchanged in the middle of the exponential phase for the same volume of deuterated ModC1 medium supplemented with 2% unlabelled glycerol (Koruza, Lafumat, Végvári *et al.*, 2018 ▸; Duff *et al.*, 2015 ▸). Upon dilution in the deuterated medium, the cells were allowed to recover for 1 h at 37°C while shaking at 120 rev min^−1^. Following the adaptation period, the temperature was decreased to 25°C and shaking was increased to 200 rev min^−1^. Protein expression was induced by the addition of IPTG in the presence of 1 m*M* zinc sulfate. The cells were harvested after 18 h by centrifugation and stored at −20°C. Deuterated CA IX_SV_ was purified as described for the protiated form in Section 2.1.

### 2.3. CA IX_SV_ crystallization optimization and H/D exchange   

Crystallization drops were prepared using both the hanging-drop and sitting-drop vapour-diffusion methods after a lengthy optimization procedure as described elsewhere (Koruza, Lafumat, Nyblom *et al.*, 2018 ▸). Briefly, crystals were initially grown for the preparation of seed stocks using a 1:1 ratio of protein solution (17 mg ml^−1^ H/H CA IX_SV_) and 30%(*w*/*v*) PEG 4000, 0.1 *M* Tris–HCl pH 8.5, 0.2 *M* sodium acetate or 0.2 *M* ammonium formate. These crystals were then sacrificed for seed-stock preparation in the mother liquor as described in the instructions for the Seed Bead Kit (Hampton Research; https://www.hamptonresearch.com). Crystallization was repeated using a 3:2:1 ratio of protein:precipitant:seed stock in drop volumes of between 6 and 24 µl. With seeding, crystals appeared within a week. Both protiated and deuterated CA IX_SV_ were crystallized using protiated buffers. Crystals of H/H CA IX_SV_ were used without further manipulation. To prepare H/D CA IX_SV_ and D/D CA IX_SV_ crystals, the reservoir solution was removed and replaced with a deuterated version. The drops were then resealed and allowed to H/D-exchange for several weeks prior to X-ray data collection. Prior to cooling the crystals by plunging them into liquid nitrogen, they were cryoprotected by dipping them into reservoir solution supplemented with 20% glycerol. For the H/D and D/D crystals, deuterated glycerol was used for cryoprotectant preparation.

### 2.4. Crystallographic data collection and structure refinement   

Two diffraction data sets for H/H CA IX_SV_ were collected at 100 K on the FIP-BM30A beamline (Roth *et al.*, 2002 ▸) at the European Synchrotron Radiation Facility (ESRF), Grenoble, France and on the BioMAX beamline at MAX IV Laboratory, Lund, Sweden. The H/D and D/D CA IX_SV_ data were collected on the BioMAX beamline at MAX IV Laboratory, Lund, Sweden.

Data processing was performed using the *autoPROC* software package (Vonrhein *et al.*, 2011 ▸). The automated workflow script mainly uses *XDS* (Kabsch, 2010 ▸) as the data-processing and scaling software and *POINTLESS* for space-group determination. For two of the data sets (H/D and D/D) 3600 images were collected. The images were processed in batches to find a cutoff where radiation damage impacts the data quality. However, data processing and subsequent model refinement showed that it was best to use the full data sets.

The phases for all of the X-ray data were obtained by molecular replacement in *Phaser* (McCoy *et al.*, 2007 ▸) using PDB entry 5dvx (Mahon *et al.*, 2016 ▸) as a search model. The models were initially rigid-body refined in *Phaser*, followed by restrained refinement in the *PHENIX* suite (Adams *et al.*, 2011 ▸). For all data sets, a bulk-solvent correction and a free *R*-factor monitor (calculated with 5% of randomly chosen reflections) were applied throughout the refinement. 2*F*_o_ − *F*_c_ and *F*_o_ − *F*_c_ map interpretation and manual model building was performed using *Coot* (Emsley *et al.*, 2010 ▸). For the apparently larger *P*2_1_ unit cell, both chains were refined without applying noncrystallographic symmetry (NCS).

Figures were generated using *PyMOL* (Schrödinger; http://www.pymol.org). The CA IX_SV_ structures were deposited in the RCSB Protein Data Bank with the following accession codes: 6rqn, 6rqq, 6rqu and 6rqw. Data-collection and refinement statistics are summarized in Table 1 ▸. Dimer-interface analysis, buried surface-area calculation and mapping of interactions were performed in *PyMOL* and *Coot* and using the *PDBePISA* server (Emsley *et al.*, 2010 ▸; Krissinel & Henrick, 2007 ▸).

## 3. Results and discussion   

### 3.1. Crystallography   

**Figure 1:**
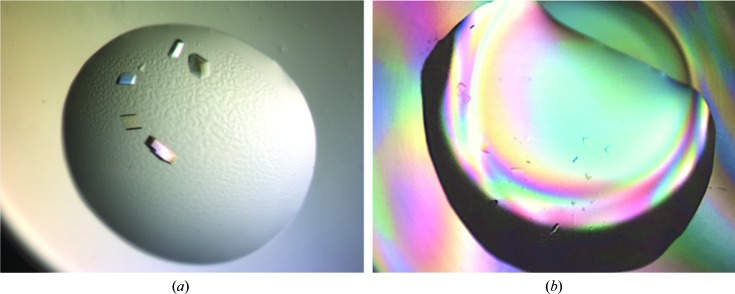
Photographs of hanging-drop and sitting-drop vapour-diffusion setups for producing (*a*) protiated and (*b*) deuterated CA IX_SV_. Both of the drops shown here are 10 µl in volume.

Crystals for X-ray data collection were obtained in both hanging-drop and sitting-drop vapour-diffusion setups. Microseeding into drop volumes varying between 3 and 10 µl produced crystals within 1–2 weeks. There were noticeable and reproducible differences in the number, size and quality of the crystals depending on the deuteration status of the protein (Fig. 1 ▸; Koruza, Lafumat, Végvári *et al.*, 2018 ▸). For the crystals used in this study the volumes ranged from 0.01 to 0.03 mm^3^. The largest CA IX_SV_ crystal that we obtained was 0.8 mm^3^ and efforts to scale up and increase the volume continue (see Fig. 5 in Koruza, Lafumat, Nyblom *et al.*, 2018 ▸). We obtained crystals in space group *P*2_1_ with two apparently different unit cells labelled ‘small’ (unit-cell parameters *a* = 44.5, *b* = 65.4, *c* = 46.7 Å, β = 115.1°) and ‘big’ (unit-cell parameters *a* = 48.9, *b* = 65.1, *c* = 76.3 Å, β = 92.86°). Data from H/H crystals were initially collected on the FIP-BM30 beamline at the ESRF and they were shown to belong to a different space group to the previously reported *P*2_1_2_1_2_1_ (Mahon *et al.*, 2016 ▸). Subsequent data collection from H/H, H/D and D/D crystals on the BioMAX beamline at MAX IV Laboratory revealed that H/H also indexed as space group *P*2_1_ but with a ‘big’ unit cell (Table 1 ▸). The other H/D and D/D crystals were both in the ‘small’ *P*2_1_ unit cell, the same as the first one we determined from ESRF data. A summary of data-set and refinement statistics is shown in Table 1 ▸. The crystals all diffracted with good statistics and the structures were determined to 1.77–1.28 Å resolution.

### 3.2. Space-group and crystal-packing analysis   

**Figure 2:**
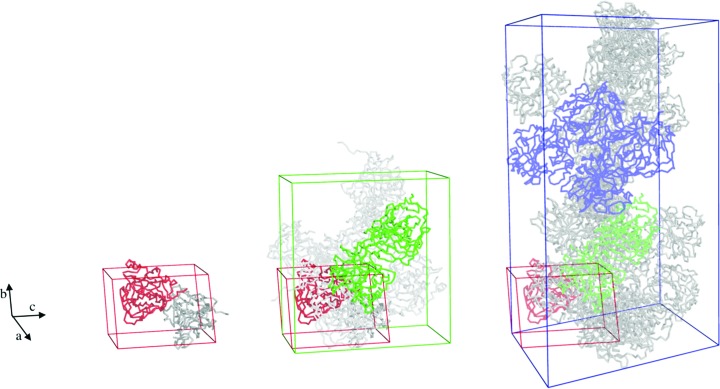
Crystal packing diagrams of CA IX_SV_ in *P*2_1_ (this work) and *P*2_1_2_1_2_1_ (PDB entry 5dvx), and CA IX in *P*6_1_ (PDB entry 3iai) unit cells. The monomer in the ASU from the small *P*2_1_ is shown as a red ribbon in all diagrams for reference. The two chains in the ASU unit of *P*2_1_2_1_2_1_ dimer are in green and in blue for *P*6_1_.

The small *P*2_1_ monoclinic unit cell contained two CA IX_SV_ chains (one per asymmetric unit) with a volume of 123 080 Å^3^. The other H/H crystal unit cell processed as a ‘big’ *P*2_1_ cell, but is in fact a doubled ‘small’ unit cell with the two chains in the apparent ASU related by translational NCS of ½, 0, ½. So, while theoretically redundant to the ‘small’ H/H structure in terms of packing and contacts, the crystallization conditions were slightly different and we do see a different ligand bound in the active site (discussed later in Section 3.3). For these reasons we show the data statistics in Table 1 ▸ and discuss the active site in Section 3.3 and Fig. 5. The CA IX_SV_ packing arrangements are shown in Fig. 2 ▸, with symmetry-related molecules shown in grey.

Previous biophysical studies of CA IX have established it to be dimeric both *in vivo* and *in vitro*, although the precise organization of the native dimer is unknown (Hilvo *et al.*, 2008 ▸; Li *et al.*, 2011 ▸). A dimer of dimers was observed in the first published crystal structure of the CA IX catalytic domain; it was produced using a baculovirus expression-vector system and the structure was determined in space group *P*6_1_ (PDB entry 3iai; unit-cell parameters *a* = *b* = 144.2, *c* = 208.9 Å; Alterio *et al.*, 2009 ▸). In this structure protein dimerization was mediated by an intermolecular disulfide bond involving Cys174 (position 41 in CA II) and was proposed to be a physiologically relevant quaternary structure. Interestingly, in the same report a Cys174Ser CA IX variant crystallized with the same packing and dimeric interface but without the disulfide bond (Alterio *et al.*, 2009 ▸). Recently, another study reported a CA IX structure, this time determined from protein expressed in yeast, that crystallized in space group *H*3 (PDB entry 6fe0; unit-cell parameters *a* = *b* = 152.9, *c* = 171.5 Å; Kazokaitė *et al.*, 2018 ▸). In addition to these studies, a crystal structure of CA IX_SV_ was also determined in space group *P*2_1_2_1_2_1_ (PDB entry 5dvx; unit-cell parameters *a* = 57.9, *b* = 102.7, *c* = 108.9 Å). This structure also had two NCS chains in the ASU, however, the arrangement did not correspond to the previous reports of the native CA IX. This was most likely owing to the Cys174 residue being mutated to a serine, but the unit cell and crystal packing were also different (Mahon *et al.*, 2016 ▸). As such, this smaller ortho­rhombic space-group unit cell was more suitable for neutron studies than the previously published hexagonal cell, and we pursued the orthorhombic form for our studies. Consideration of the unit-cell parameters in neutron protein crystallography experiments (normally up to a maximum of 150 Å) is related to the limitations of the current flux of neutron sources as well as the layout of macromolecular beamlines to resolve larger unit-cell parameters (Meilleur *et al.*, 2018 ▸; O’Dell *et al.*, 2016 ▸). To be able to obtain reasonable diffraction data (better than 2 Å resolution) from a crystal with a large unit cell, it is necessary to also optimize the overall crystal volume (Tanaka, 2019 ▸). Despite extensive efforts to reproduce these crystals of CA IX_SV_, we instead obtained two new and seemingly different monoclinic *P*2_1_ crystals (Fig. 1 ▸, Table 1 ▸). The ‘small’ monoclinic *P*2_1_ crystal form is isomorphous to the deuterium-labelled CA IX_SV_ crystal form. In addition, there is only one CA IX_SV_ per asymmetric unit, which is useful when using neutrons for protein crystallography (Blakeley *et al.*, 2015 ▸). For a complete list of crystal contacts, refer to Table 2 ▸.

**Figure 3:**
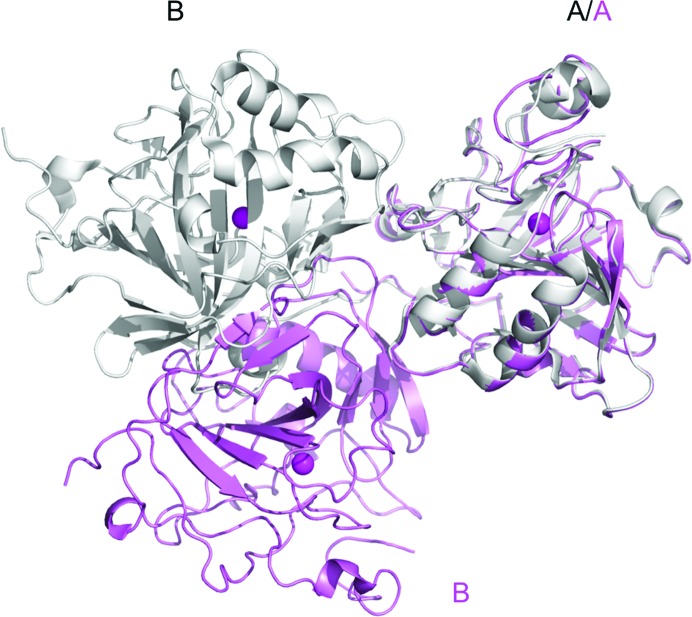
Overlay of the two chains in the unit cell of *P*2_1_ (this work) and the two chains in the ASU of *P*2_1_2_1_2_1_ (PDB entry 5dvx; Mahon *et al.*, 2016 ▸) with chain *A* as reference to illustrate differences in crystallographic organization. The chains for the small *P*2_1_ unit cell are shown as a pink cartoon while the two NCS chains in the ASU from *P*2_1_2_1_2_1_ are shown as a grey cartoon. Zinc atoms are shown as magenta spheres to indicate the location of the active site.

Fig. 3 shows an overlay of the two chains in the monoclinic unit cell with the two chains in the orthorhombic ASU, with chain A from the latter as the reference. The monomer-to-monomer electrostatic contacts for both are listed in Table 2 ▸. There are more interactions mediating the interface in the orthorhombic chains, most of which are attributable to the C-terminal residues wrapping around the dimer pair. The observation of multiple monomer-to-monomer arrangements suggests that CA IX has a strong propensity to dimerize, independent of intermolecular disulfide bonds, and it is not possible to infer which may be the relevant physiological dimer. The differences in arrangement and crystal packing between native CA IX and CA IX_SV_ are illustrated in Fig. 2 ▸.

**Figure 4:**
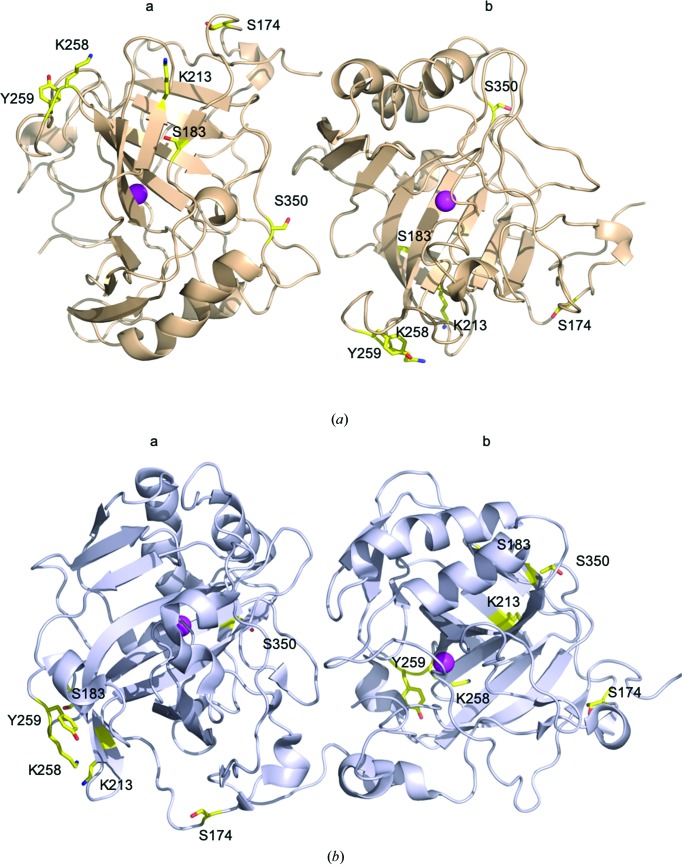
Comparison of crystallographic contacts in view of the locations of the six mutations present in CA IX_SV_. CA IX_SV_ is shown in cartoon representation, substituted amino acids (compare with CA IX) are depicted as yellow sticks, zinc is shown as a magenta sphere. (*a*) Crystallographic chains in the small *P*2_1_ unit cell (this work), (*b*) NCS chains in the ASU of the *P*2_1_2_1_2_1_ cell (PDB entry 5dvx; Mahon *et al.*, 2016 ▸).

The CA IX_SV_ variant has six surface amino-acid substitutions compared with CA IX, which were chosen to optimize expression in *E. coli* and crystallization. The intention was to eliminate dimerization owing to disulfide-bond formation, to reduce surface hydrophobicity and to encourage possible crystal contacts based on CA II crystal contacts (Mahon *et al.*, 2016 ▸). Hence, we wanted to investigate whether some of the amino-acid interactions involved in the crystal contacts in the *P*2_1_ or the *P*2_1_2_1_2_1_ unit cell were affected by the six amino-acid substitutions. Figs. 4 ▸(*a*) and 4 ▸(*b*) shows the dimer in ribbon representation, with the substituted side chains depicted as sticks. From inspection of these structures, it was apparent that residues Lys258 and Tyr259 were involved in NCS dimerization in the big monoclinic *P*2_1_ unit cell reported here [Table 2 ▸, Fig. 4 ▸(*b*)]. In the small monoclinic *P*2_1_ and previously reported orthorhombic *P*2_1_2_1_2_1_ unit cells [Fig. 4 ▸(*c*)] none of the amino-acid substitutions were involved in crystal contacts or NCS dimerization. It would therefore appear that the six surface residues that were changed do not drive dimerization but do have an important impact on the solubility and stability of the catalytic domain of CA IX.

### 3.3. Active-site comparison of H/H (small), H/H (big), HD and DD CA IX_SV_ structures   

**Figure 5:**
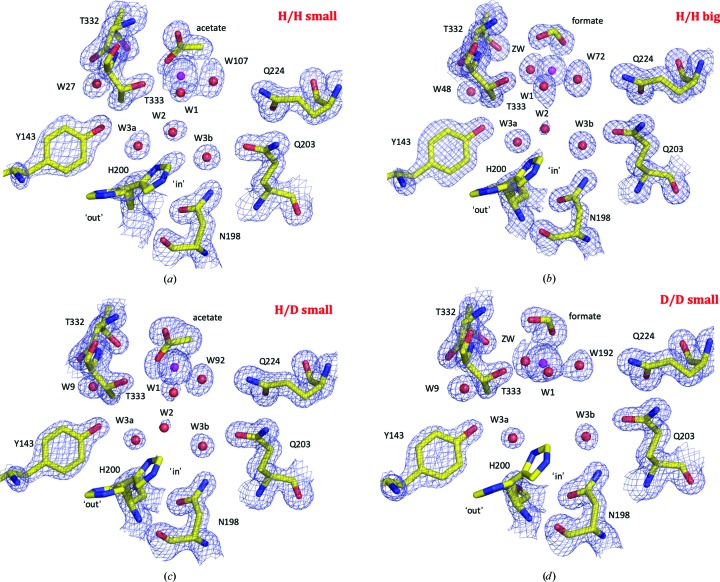
Active-site comparison of CA IX_SV_. (*a*) H/H in the small *P*2_1_ unit cell, (*b*) H/H in the doubled *P*2_1_ unit cell, (*c*) H/D exchanged in the small *P*2_1_ unit cell and (*d*) D/D CA IX_SV_ in the small *P*2_1_ unit cell. Active-site residues are depicted as yellow sticks; water molecules and Zn atoms are shown as red and magenta spheres, respectively. 2*F*_o_ − *F*_c_ electron-density maps are shown in blue mesh and are contoured at 1.50σ for residues, 1.25σ for solvent and 3.50σ for zinc.

When deuterating proteins for neutron studies, it is important to determine whether deuteration causes appreciable conformational effects in the resulting protein side chains and active-site solvent positioning. For the deuterated protein to be useful in structural studies it has to be representative of the physiological protiated protein: there should be no conformational changes. As noted before, the overall active-site arrangement of solvent and amino-acid residues appears to be largely unaffected, with an r.m.s.d. variation for all atoms when superimposing all four structures onto each other of less than 1 Å. We conducted a careful OMIT map analysis of the structures and found that in all four structures there are one of two ions bound to the zinc ion that come from the crystallization conditions: either acetate or formate (Fig. 5 ▸). Both formate and acetate are known inhibitory anions that bind to CAs and therefore their presence is not a surprise (Coleman, 1967 ▸; Håkansson *et al.*, 1992 ▸; Alterio *et al.*, 2009 ▸). Formate inhibits by displacing the so-called ‘deep water’ in the active site and binds in the same location as the carbon dioxide substrate (Fig. 5 ▸; Domsic & McKenna, 2010 ▸), whereas acetate replaces two waters, the deep water and the catalytic zinc-bound water, effectively presenting an inhibited active-site structure (Supplementary Fig. S1). We observed formate bound in the apparent ‘big’ cell in H/H and the D/D structures, while acetate was present in the small H/H and the H/D-exchanged structures (Fig. 5 ▸). The presence of either ion does not seem to affect the overall active-site side-chain conformations, with the exception of small water rearrangements, which are reflected in the relative weak density for the active-site solvent molecule W2 [Fig. 5 ▸(*a*)]. In Figs. 5 ▸(*b*)–5 ▸(*d*) it can be seen that W2 has poor density at the same contouring and also has a higher refined crystallo­graphic *B* factor com­pared with the other active-site solvent molecules. The weak density of W2 is most apparent in the D/D structure, where there is no density below the 1.5σ level in the OMIT electron-density map. There are multiple possible explanations for this, including the presence of formate that may perturbate the water network or subtle effects caused as a result of protein deuteration.

Careful electron-density OMIT analysis of the proton-shuttling residue His200 shows it to be split between two conformations, termed the ‘in’ and the ‘out’ conformation in the CA II literature (Supplementary Fig. S2; Nair & Christianson, 1991 ▸; Fisher *et al.*, 2005 ▸). In the structure with PDB code 5dvx (CA IX_SV_) the His200 side chain was fully in the ‘out’ position and was π-stacked with Trp141 (Mahon *et al.*, 2016 ▸). There is structural evidence that the preferred position of His64 in CA II is strongly affected by the pH, with the ‘out’ conformation being dominant at low pH owing to charge repulsion between the charged His and the zinc (Fisher *et al.*, 2005 ▸). However, all the monoclinic structures reported here and the previously reported ortho­rhombic forms were all determined from crystals grown at pH 8.5, yet the alternate conformation occupancy for His200 is different (Mahon *et al.*, 2016 ▸). However, our crystals do contain either formate or acetate and the presence of these ligands could be a disrupting factor by altering the electrostatic effect of the zinc charge on the orientation of His64.

Taking the above observations together, for the four structures of the different labelled variants of CA IX_SV_ we can conclude that deuteration had little to no effect on the overall structure. This is in contrast to other parameters, such as thermal stability and crystallization behaviour, as reported previously (Koruza, Lafumat, Végvári *et al.*, 2018 ▸). Furthermore, comparing specific residues that compose the active site shows that the overall architecture is maintained between H/H, H/D and D/D CA IX_SV_.

## 4. Conclusions   

Here, we report four crystal structures of different protium/deuterium-labelled versions of CA IX_SV_ in preparation for future neutron crystallographic studies. Despite efforts to reproduce the previously published *P*2_1_2_1_2_1_ crystal form, we instead obtained a different *P*2_1_ crystal form from that previously observed for native CA IX and CA IX_SV_. This was an unexpected but fortuitous result, as the unit cell is much smaller than all previously reported for CA IX or CA IX_SV_. Overall, the optimized crystallization condition and resulting crystal parameters are more tractable for neutron studies. In addition, the structural comparison of the protiated, partially deuterated and perdeuterated crystal structures reveal that there are insignificant changes owing to deuteration. Hence, the small unit cell *P*2_1_ crystals of CA IX_SV_ will be used in future neutron crystallographic structural studies for the design of CA IX-specific inhibitors.

## Supplementary Material

PDB reference: human carbonic anhydrase IX, protiated, 6rqn

PDB reference: 6rqq

PDB reference: H/D-exchanged, 6rqu

PDB reference: deuterated, 6rqw

Supplementary Figures. DOI: 10.1107/S2059798319010027/lp5041sup1.pdf

## Figures and Tables

**Table 1:** Data-collection and model-refinement statistics for CA IX_SV_

	Protiated (H/H) (small unit cell)	Protiated (H/H) (big unit cell)	H/D-exchanged (H/D)	Deuterated (D/D)
PDB code	6rqn	6rqq	6rqu	6rqw
Source	FIP-BM30, ESRF	BioMAX, MAX IV Laboratory	BioMAX, MAX IV Laboratory	BioMAX, MAX IV Laboratory
Wavelength (Å)	0.979	0.979	0.979	0.979
Detector	ADSC Q315r	Dectris EIGER 16M	Dectris EIGER 16M	Dectris EIGER 16M
Rotation range per image (°)	0.5	0.5	0.1	0.1
Total No. of images	270	360	3600	3600
Space group	*P*2_1_	*P*2_1_	*P*2_1_	*P*2_1_
Unit-cell parameters (Å, °)	*a* = 44.3, *b* = 65.1, *c* = 46.7, β = 115.1	*a* = 48.9, *b* = 65.1, *c* = 76.3, β = 92.86	*a* = 44.5, *b* = 65.4, *c* = 46.7, β = 115.1	*a* = 44.4, *b* = 65.1, *c* = 46.6, β = 114.7
Unit-cell volume (Å^3^)	121830	241730	121830	121830
Resolution range (Å)	50.0–1.77 (1.88–1.77)	49.5–1.28 (1.29–1.28)	40.0–1.39 (1.42–1.39)	40.0–1.49 (1.51–1.49)
Total No. of reflections	63296 (9199)	422779 (20704)	327912 (15926)	265165 (13184)
No. of unique reflections	22187 (3463)	122208 (5995)	48352 (2406)	39461 (1948)
Multiplicity	2.8 (2.6)	3.5 (3.5)	6.8 (6.6)	6.7 (6.8)
Completeness (%)	95.1 (92.9)	98.4 (96.0)	99.8 (99.1)	99.9 (99.3)
〈*I*/σ(*I*)〉	10.5 (2.2)	13.1 (2.1)	19.9 (2.2)	14.3 (2.2)
*R*_merge_ † (%)	6.3 (48.6)	3.1 (48.5)	3.6 (78.1)	6.0 (78.1)
*R*_meas_ (%)	7.7 (60.3)	4.5 (67.0)	4.3 (96.2)	5.9 (86.4)
CC_1/2_ (%)	99.6 (83.3)	99.9 (86.6)	99.9 (82.2)	99.8 (69.7)
R.m.s.d., bond lengths (Å)	0.007	0.007	0.006	0.015
R.m.s.d., bond angles (°)	0.888	0.982	0.957	1.344
*R*_cryst_ ‡	0.169	0.179	0.173	0.173
*R*_free_ §	0.206	0.195	0.188	0.203
No. of solvent molecules	253	682	262	217
Mean *B* factors (Å^2^)				
Protein	26.0	21.7	29.0	29.3
Solvent	36.12	34.6	37.5	38.3
Acetate ligand	31.92		33.57	
Formate ligand		26.7		37.3

† *R*_merge_ = 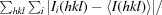 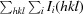 × 100.

‡ *R*_cryst_ = 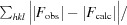 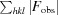.

§ *R*_free_ is calculated in the same way as *R*_cryst_ but for data omitted from refinement (5% of reflections for all data sets)

**Table 2:** Crystallographic interchain interactions in CA IX_SV_: comparison between *P*2_1_ (this work) and *P*2_1_2_1_2_1_ (PDB entry 5dvx). Hydrogen-bond and salt-bridge distances are shown in parentheses and indicate donor–acceptor (heavy atom) distances. *A* chains are listed first. Interactions longer than 3.4 Å were excluded. Analysis was performed using the *PBDePISA* server and was verified visually in *Coot* (Krissinel & Henrick, 2007 ▸; Emsley *et al.*, 2010 ▸). Mutated residues in CA IX_SV_ are shown in bold.

*P*2_1_ ‘small’ (this work): monomer in asymmetric unit	*P*2_1_ ‘big’ (this work): dimer in asymmetric unit	*P*2_1_2_1_2_1_ (PDB entry 5dvx): dimer in asymmetric unit
Glu280–Glu192 via water (2.6 and 2.8 Å)	Glu297–Glu219 via water (2.8 and 2.7 Å)	Arg167–Asp146 (–CO) (3.1 Å)
Gln307–Arg323 (2.9 Å)	Glu297–Thr257 via water (2.8 and 2.7 Å)	Gln169–Pro148 (–CO) via water (2.7 and 2.8 Å)
Ser319–Glu305 (2.7 Å)	Glu301–**Lys258** (–CO) (3.4 Å)	Gly233 (N)–Glu305 (–CO) (2.8 Å)
Asp320–Gln307 (2.9 Å)	His357– Pro175 via water (3.0 and 3.4 Å)	Glu242–Trp141 (N) (3.2 Å)
Arg323–Glu298 (3.2 Å)	Asp361–Pro216 (–CO) (3.3 Å)	Gly243 (–CO)–Gly367 (N) (2.9 Å)
Arg323–Thr306 (3.2 Å)	Asp368–Arg268 via water (2.5 and 3.1 Å)	His244–Zn–His200 (3.3 Å)
Asn346–Glu192 (3.1 A)	Asp368–**Lys258** (2.7 Å)	His244–Zn–Trp141 (N) (3.3 Å)
Asn346–Gln307 (2.8 Å)	Asp368–**Tyr259** via water (3.2 and 3.0 Å)	His244–Glu302 (2.8 Å)
Gln347–Glu305 (2.8 Å)		Arg245 (N)–Glu302 (2.9 Å)
		Asp395 (–CO)–Val152 (N) (2.9 Å)
		Ser396–Ser153 via water (2.6 and 2.6 Å)
		Arg399–Asp263 (–CO) (2.8 Å)
		Arg399–Leu266 (–CO) (2.7 Å)
