# Supplementary material for: Structural comparison of protiated, H/D-exchanged and deuterated human carbonic anhydrase IX
Source: Acta Crystallogr D Struct Biol. 2019 Aug 22;75(Pt 10):895–903. doi: 10.1107/S2059798319010027 (PMC6778848; doi:10.1107/S2059798319010027)
Supplement: Supplementary file 1 [file d-75-00895-sup1.pdf]

STRUCTURAL BIOLOGY

Volume 75 (2019)

Supporting information for article:

Structural comparison of protiated, H/D-exchanged and deuterated human carbonic anhydrase IX

K. Koruza, B. Lafumat, M. Nyblom, B. P. Mahon, W. Knecht, R. McKenna and S. Z. Fisher

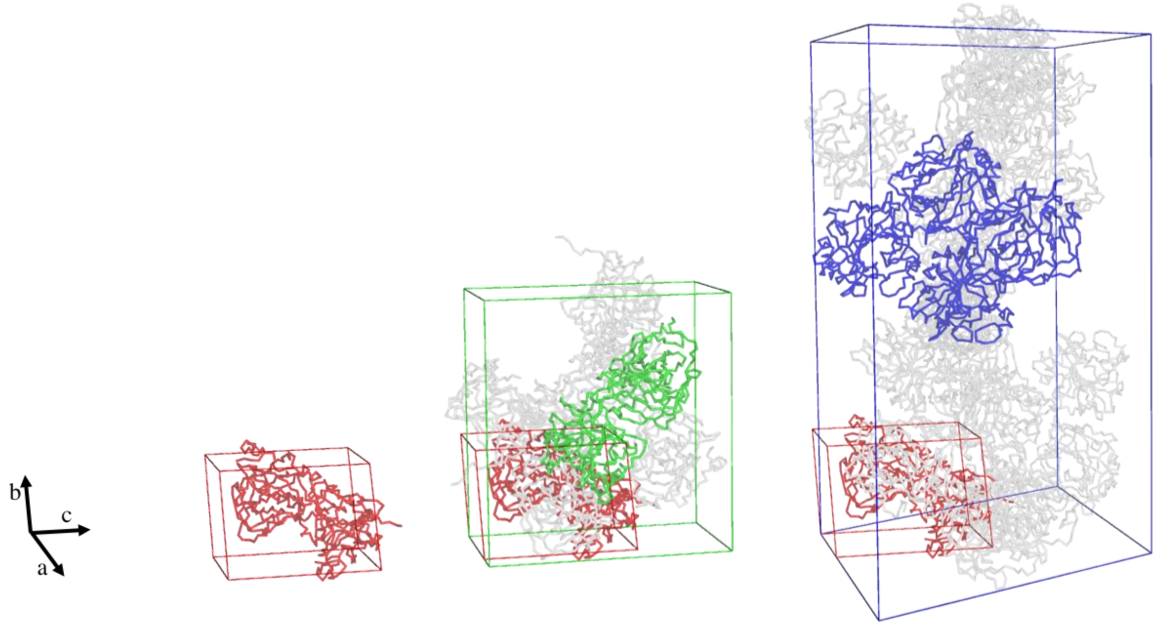

Figure S1 Crystal packing diagrams of the small $P2_1$ (this work), $P2_12_12_1$ (5dvx), and $P6_1$ (3iai) unit cells for CA IX. The monomer from the small $P2_1$ is shown as a red ribbon in all diagrams for reference. The $P2_12_12_1$ dimer is in green and the $P6_1$ tetramer is in blue.

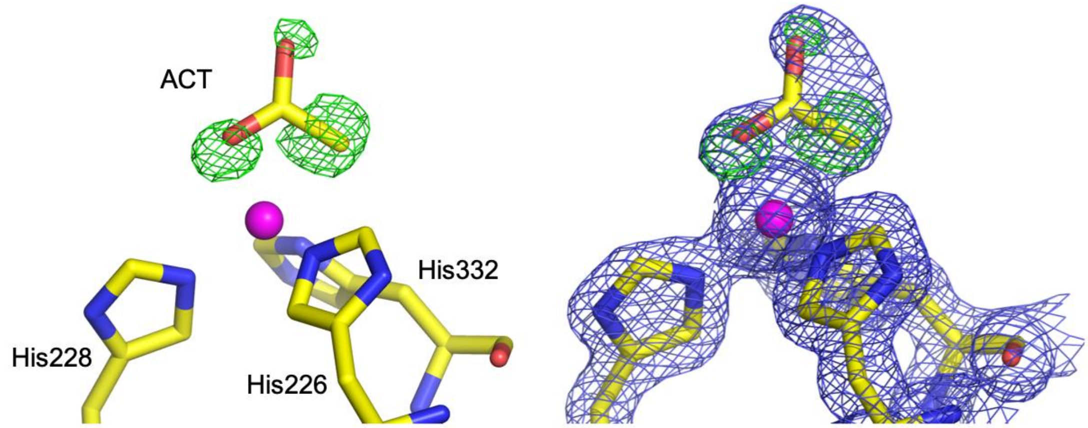

Figure S2 Omit density of acetate bound to the active site Zn in CA IX_{SV}. The F_o-F_c maps is shown in green mesh, contoured at 6σ, $2F_o$-F_c is shown in blue mesh and is contoured at 1.5σ. Zn is shown as a magenta sphere, Zn ligand His residues as labeled.

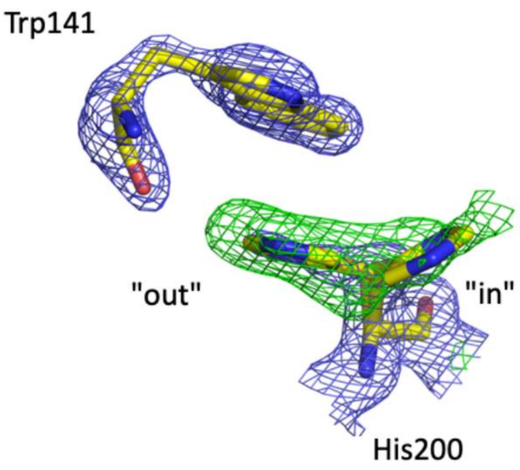

Figure S3 Omit density of His200 side chain in CA IX_{SV} shows disorder between "in" and "out" conformation. The "out" conformation is making a pi-stack with Trp141. The F_o-F_c maps is shown in green mesh, contoured at 2σ, 2F_o-F_c is shown in blue mesh and is contoured at 1.5σ.
